# Exploring the impact of legume consumption on undernutrition in rural Malawian children aged 6–59 months old: a community-based cross-sectional study

**DOI:** 10.1017/jns.2025.10073

**Published:** 2026-01-13

**Authors:** Patrick Ndovie, Numeri Chalumpha Geresomo, Smith G. Nkhata, Robert Fungo, Vincent Nyau, Justice Munthali

**Affiliations:** 1 Department of Human Nutrition and Health, Lilongwe University of Agriculture and Natural Resources, Lilongwe, Malawi; 2 Department of Agriculture and Food Systems, Natural Resources College, Lilongwe University of Agriculture and Natural Resources, Lilongwe, Malawi; 3 School of Food Technology, Nutrition & Bioengineering, Makerere University, Kampala, Uganda; 4 School of Agricultural Sciences, Department of Food Science & Nutrition, University of Zambia, Lusaka, Zambia; 5 Alliance of Bioversity International & CIAT, Malawi, Agricultural Research Station, Lilongwe, Malawi

**Keywords:** Dietary diversity, Legume consumption, Malnutrition, Micronutrients, Rural Malawi, AOR, Adjusted Odds Ratio, CI, Confidence Interval, CIAT, Alliance of Bioversity International & CIAT, HAZ, Height-for-Age Z-score, MK, Malawian Kwacha, MUAC, Mid-Upper Arm Circumference, MSU, Michigan State University, NHSRC, National Health Sciences Research Committee, NSO, National Statistical Office, SSA, Sub-Saharan Africa, SD, Standard Deviation, UNICEF, United Nations International Children’s Emergency Fund, WHO, World Health Organization, WAZ, Weight-for-Age Z-score, WHZ, Weight-for-Height Z-score

## Abstract

Malnutrition remains a major public health issue in Sub-Saharan Africa, with one-third of all malnourished children residing in the region. In Malawi, 37.1% of children under five are stunted, and 63% are anaemic. Poor diets and poverty contribute significantly. Legumes, being rich in protein, fibre, and micronutrients, offer a sustainable food-based approach to improve child nutrition and support local agriculture. This study aimed at assessing the association between legume consumption and nutritional status in children aged 6–59 months in rural Malawi. A community-based cross-sectional study was conducted in Mzimba, Mchinji, and Mangochi districts, involving 1275 children. Data were collected on dietary intake, socioeconomic status, and anthropometry using semi-structured questionnaires. Nutritional status was determined using WHO Anthro, and associations were analysed using logistic regression in Stata. Prevalence of stunting was 42.8%, underweight 17.4%, and wasting 8.4%. Over half of the children did not consume legumes. Pigeon pea consumption significantly reduced odds of wasting (AOR = 0.14), and common beans were associated with lower odds of both wasting and stunting. Conversely, groundnut consumption was linked to increased underweight (AOR = 1.68). Animal food consumption was associated with lower underweight but higher odds of wasting. Legume consumption showed both protective and adverse associations with child malnutrition. In conclusion, this study has shown that promoting dietary diversity and appropriate legume use could enhance nutrition outcomes. Findings highlight the potential of legumes in addressing undernutrition but also the need for targeted nutrition education and interventions in rural Malawi.

## Introduction

Malnutrition poses a significant public health challenge, more especially on children, in developing countries.^(1)^ It is one of the leading cause of mortality and morbidity affecting millions of children globally.^(2)^ Sub-Saharan Africa bears a considerable burden, with approximately one-third of malnourished children worldwide, including 39% stunted, 10% wasted, and 25% underweight children under five years old.^(3)^ In Malawi, malnutrition remains a pressing issue, notably with a reported stunting prevalence of 37.1%, surpassing the 20% threshold set by the World Health Organization (WHO). In addition to high stunting levels, Malawi has high prevalence rate of anaemia which is at 63%.^(4,5)^ Malawi, predominantly occupied by rural population accounting for 84% depends on subsistence agriculture.^(6)^ Furthermore, NSO (Malawi) report of 2015–2016 revealed that stunting levels were fairly distributed across the regions with northern, central and southern regions accounting for 35%, 38% and 37% respectively.^(7)^ One of the leading causes of malnutrition is poverty which leads to difficulties in accessing nutritious and quality diets for children. According to Malawi poverty report in 2020, 56.5% of the rural population is poor. In addition, at region level, northern, central and southern regions had 35.9%, 62.8% and 56.7% of the population were poor respectively.^(8)^

The dietary patterns that are established during early childhood play a crucial role in promoting optimal growth and development in children.^(9)^ Inadequate dietary intakes among children under the age of five can lead to developmental impairments, resulting in susceptibility to illnesses and mortality during childhood, and may pose lasting health challenges into adulthood. Legumes provide such an opportunity, as their protein content is significantly higher than cereals, and they are rich in dietary fibre, starch, minerals, vitamins, and antioxidants.^(10)^ Legumes are an important part of the diet in sub-Saharan Africa (SSA), providing a valuable source of protein, fibre, vitamins, and minerals. They are widely cultivated by smallholder farmers and play a key role in food and nutrition security, income generation, and sustainable intensification of agriculture in the region. The major grain legumes grown in SSA include cowpea, common bean, groundnut, soybean, pigeon pea, and chickpea.^(11,12)^

In Malawi, studies have tested legumes such as cowpea and common bean in complementary foods, showing improvements in child growth and reduced gut dysfunction.^(10,13)^ Population-level analyses of the 2015–16 Malawi DHS indicate appropriate complementary feeding, which may include legumes, is linked to lower stunting risk.^(13)^ Across sub-Saharan Africa, legume production supports dietary diversity, though effects on growth are less clear.^(14)^ Recent assessments of legume-based flour blends in Malawi and Zambia reveal variability in nutrient content.^(15)^ Despite these findings, few studies evaluate the prevalence of legume consumption among children and its direct association with growth outcomes.

Given the crucial role of early childhood nutrition in supporting growth and preventing long-term health issues, it is essential to explore the dietary patterns that contribute to optimal development. While legumes offer a promising nutritional solution due to their high protein and micronutrient content, their consumption among young children in Sub-Saharan Africa remains suboptimal. This study aims to assess the prevalence of legume consumption among children aged 6–59 months in rural Malawi and investigate its potential associations with nutritional outcomes such as stunting, wasting, and underweight. By examining these patterns, the study seeks to provide insights into improving dietary practices and reducing malnutrition in the region.

## Methods

### Study area

**Figure 1.**
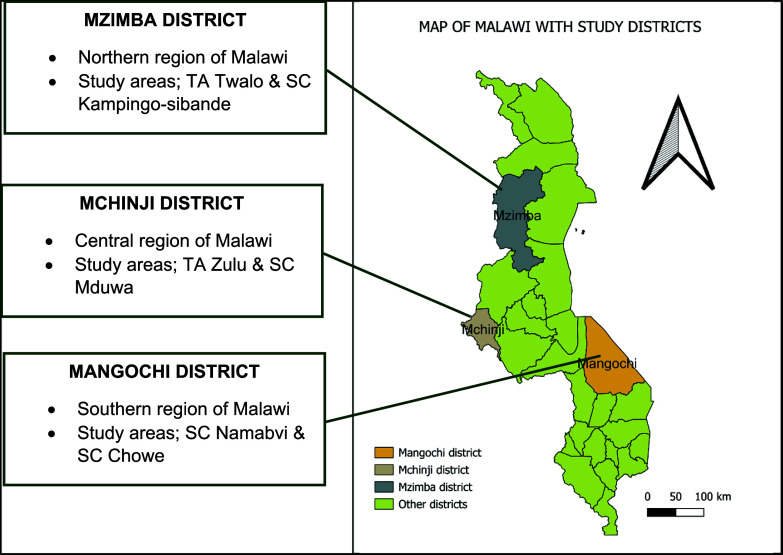
Map of Malawi highlighting the study areas where data on legume consumption and undernutrition among children aged 6–59 months was collected.

The study was conducted in Mzimba (TA Twalo & SC Kampingo-sibande), Mchinji (TA Zulu & SC Mduwa) and Mangochi (SC Namabvi & SC Chowe) Districts of Malawi (Figure 1). The three districts represented the three regions in Malawi and purposively selected due to high stunting rates within each respective region. (Mzimba – 38.9%, Mchinji – 44% and Mangochi – 45.4%).^(7)^

### Study design and population

#### Study design

A descriptive cross-sectional study was undertaken in rural households across the Northern, Central and Southern regions. A total of 1275 children, including both breastfeeding and non-breastfeeding children aged 6 to 59 months, were purposefully selected for inclusion in the study (Mzimba in the Northern region = 372, Mchinji in the Central region = 426 and Mangochi in Southern region = 478). A multistage purposive sampling approach was employed to ensure diversity and representativeness across the three selected districts: Mzimba (northern region), Mchinji (central region), and Mangochi (southern region). Within each district, Traditional Authorities (TAs) and villages were purposively selected based on accessibility, population size, and the predominance of legume consumption. From each selected village, households with at least one child aged 6–59 months were identified using community health registers, after which systematic random sampling was applied to select eligible participants. This approach ensured inclusion of households from varied socioeconomic and agricultural backgrounds while minimising selection bias through partial randomisation at the household level. The sample size was determined using a formula outlined by Pourhoseingholi, Vahedi, Rahimzadeh^(16)^:

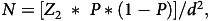




where

*N* represents the required population size,

*Z* denotes the 95% confidence level (with a standard value of 1.96),

*P* denotes the proportion of children under 5 years from the three regions based on data from the 2018 Malawi population and housing census,^(17)^ and

*D* denotes precision at 5%

#### Study population

The study population consisted of caregivers and their children aged 6–59 months, selected from households in sampled villages. Eligible participants were those who had resided in the study area for more than one year prior to the survey and provided written informed consent. Exclusion criteria applied to caregivers who either declined to consent or based on medical records or child health cards, were identified as mentally challenged, physically deformed, or chronically ill, as these conditions could influence anthropometric outcomes and require specialised feeding practices.

**Figure 2.**
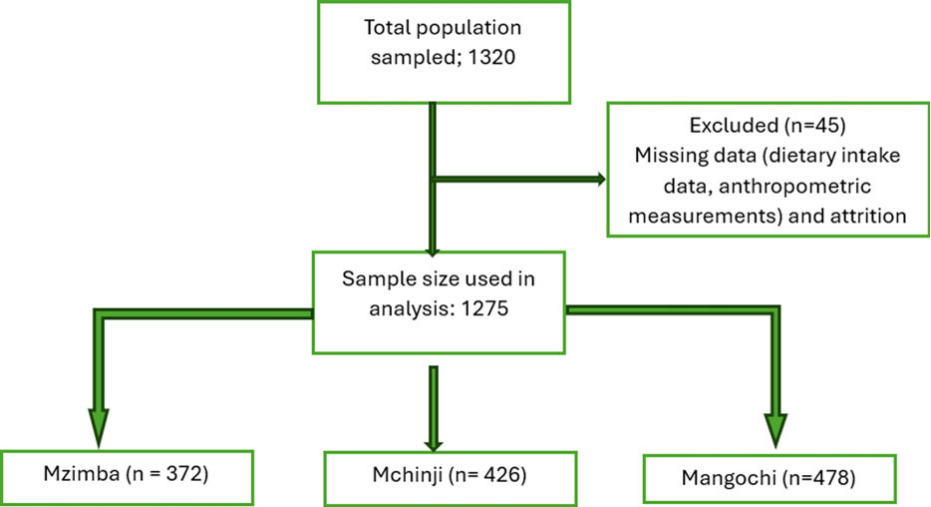
Flowchart depicting participant selection and data inclusion for the study assessing the impact of legume consumption on nutritional status among children aged 6–59 months in rural Malawi.

Figure 2 illustrates the process of participant selection leading to the final sample of 1275 children. Of the 1320 participants initially enrolled, 45 (3.4%) were excluded due to incomplete dietary intake information, missing anthropometric data, or inconsistencies in age verification. Therefore, a total of 1275 participants with complete data on key variables were included in the final analysis. Missing data were minimal (<5%) and were handled through listwise deletion, as their exclusion did not significantly alter the sample’s demographic or socioeconomic distribution.

### Socioeconomic and demographic characteristics

Socio-economic and demographic data such as sex, age (months) and gender of the children and education level, economic activities, marital status, and religion of the caregiver was collected using a semi-structured questionnaire through face-to-face interview. Additionally, data on household characteristics such as household income and size was collected using the same questionnaire. The semi-structured questionnaire was uploaded into SurveyCTO Collect v. 2.80^(18)^ platform and enumerators used electronic gadgets with SurveyCTO Collect v. 2.80 during data collection.

### Legume consumption pattern

A semi-structured questionnaire was also used to collect data on consumption frequency of legumes and legume-based food products by 6–59 months old children. The dataset also captured whether each child was breastfed, non-breastfed, or formula-fed at the time of the survey. This variable was integrated into the dietary assessment to distinguish differences in intake patterns arising from feeding type. Caregivers reported the legume and legume-based product that was given to their children 24 hours prior to the study survey. The study relied solely on the 24–hour dietary recall from the caregiver/mother as such intakes recorded only as presence or absence of consumption. Additionally, caregivers were asked to report on the legume and legume-based food consumption frequency thus how many times do they feed a particular legume or legume-based food product per week and/or per month to provide contextual information on habitual intake patterns.

### Anthropometric data

Anthropometric data was collected to assess the prevalence of nutritional status indicators (such as underweight, wasting, and stunting) among children aged 6–59 months old. Each child’s measurements included weight, and height. Children were weighed in light attire and barefoot using a calibrated portable electronic digital scale accurate to 0.1kg. For children aged less than 2 years, recumbent length was measured using UNICEF infantometer, while for children aged 2 years and above, standing height was measured using a UNICEF stadiometer to the nearest centimetre (cm). Weight was measured with a calibrated portable electronic digital scale accurate to 0.1kg. Weight and height/length measurements were taken twice, with a third measurement conducted if there was a difference of more than 0.2 kg, for weight or greater than 0.3cm for height/length and the average weight and height/length was recorded.

### Data analysis

Stata version 17.0 (Standard edition) was utilised to compute descriptive statistics including means, standard deviations (SD), frequencies, and percentages. Both univariate and multivariate logistic regression analyses were conducted to examine the relationships between food group consumption, particularly legumes and nutritional status indicators (wasting, stunting, and underweight). The statistical significance was determined at a 95% confidence level (*P* < 0.05).

Anthropometric data were processed using WHO Anthro version 3.2.2 software to calculate the child’s height-for-age z-scores (HAZ), weight-for-age z-scores (WAZ), and weight-for-height z-scores (WHZ) using the WHO growth standards reference data.^(19)^ The Z-Scores were then exported into Stata for further analysis, incorporating age-specific cut-off points to classify nutritional status categories (severely underweight, moderately underweight, normal, overweight, and obese). In the multivariate logistic regression analysis model, potential confounding variables were controlled to ensure more accurate estimates of association. The covariates included the child’s age (in months), sex, recent illness (fever, and diarrhoea within two weeks prior to the survey), caregiver’s education level, household income, household size, and district of residence. These variables were selected based on theoretical relevance from previous literature indicating their influence on child nutritional outcomes. The adjusted odds ratio (AOR) with 95% confidence interval (CI) were reported to determine the strength and direction of associations between dietary factors including legumes and legume-based food products and child nutritional status.

### Ethical considerations

This study was conducted in full accordance with the ethical principles outlined in the Declaration of Helsinki. The research protocol, including the tools used for data collection, was reviewed and approved by the National Health Sciences Research Committee (NHSRC) Ethics Committee (Approval number 23/01/4301). District councils from the three districts also approved the study, and the confidentiality and significance of the research were clearly explained to all participants. Verbal informed consent was obtained from all respondents involved in the study.

## Results

### Characteristics of caregivers and children

Characteristics of the 1275 caregivers/children who participated in the study are presented in Table 1. There were 48.3% and 51.7% males and females respectively. The mean ± SD age of the children was 28.42 ± 15.10 months, with 8.4%, 42.8% and 17.4% being wasted, stunted and underweight respectively. Most children (89.6%) aged 6–11 months old were still being breastfed, while 10.4% were not. Approximately 81.9% of children did not experience diarrhoea in the two weeks leading up to the survey, while 47.5% showed no symptoms of fever.

**Table 1.** Descriptive characteristics of the caregivers and children aged 6–59 months old included in the study (*N* = 1275)

Variable	Mean ± SD and frequency (%)
Children characteristics	
Age (months)	28.42 ± 15.10
Sex of Child	
Male	616 (48.3)
Female	659 (51.7)
Prevalence of undernutrition	
Wasting (WHZ < -2SD)	105 (8.4)
Stunting (HAZ < -2SD)	546 (42.8)
Underweight (WAZ<-SD)	218 (17.4)
Breastfeeding (6–11 months)	
Yes	59 (4.6)
No	1216 (95.4)
Diarrheal (past 2wks)	
Yes	231 (18.1)
No	1044 (81.9)
Fever (past 2wks)	
Yes	670 (52.5)
No	605 (47.5)
Fully vaccinated	
Yes	644 (50.5)
No	631 (40.5)
Caregiver’s characteristics	
Occupation	
Farmer	921 (72.2)
Permanent employee	16 (1.3)
Casual labourer	8 (0.6)
Business/trader	93 (7.3)
Housewife	86 (6.8)
Student	21 (1.7)
Unemployed	130 (10.2)
Education	
None	23 (1.8)
Junior primary education	473 (37.1)
Senior primary education	468 (36.7)
Junior secondary education	143 (11.2)
Senior secondary education	155 (12.2)
University diploma	8 (0.6)
University degree	5 (0.4)
Marital status	
Single	66 (5.18)
Married	1058 (83.0)
Divorced	66 (5.2)
Separated	9 (0.7)
Widowed	76 (6.0)
Household income (MK)	
< 50,000 (<$28.79** USD)	1192 (93.5)
≥ 50,000 (≥$28.79** USD)	83 (6.5)
Household Size	
< 4 members	516 (40.5)
≥ 4 members	759 (59.5)
Religion	
Christian	791 (62.0)
Muslims	479 (37.6)
No religion	5 (0.4)

* WHZ weight-for-age z-scores, HAZ height-for-age z-scores, WAZ weight-for-age z-scores, SD Standard deviations **$1 = MK1,737.00.00.

Furthermore, 50.5% of the caregivers indicated that their children received all the vaccines prior to the study, with 72.2% of the caregivers being farmers and the least being casual labourers (0.6%). About 37.1% of the caregivers had attained junior primary education and 83% and 0.4% were married and separated respectively. Furthermore, 93.5% of the households earned less than 50,000mk **(28.79 USD)** per month, and 59.2% of the households had greater than 4 members. On religious affiliations, 62.0% of the caregivers were Christians, while 0.4% had no religious affiliation.

### Legume and legume-based consumption among 6–59 months old children

**Figure 3.**
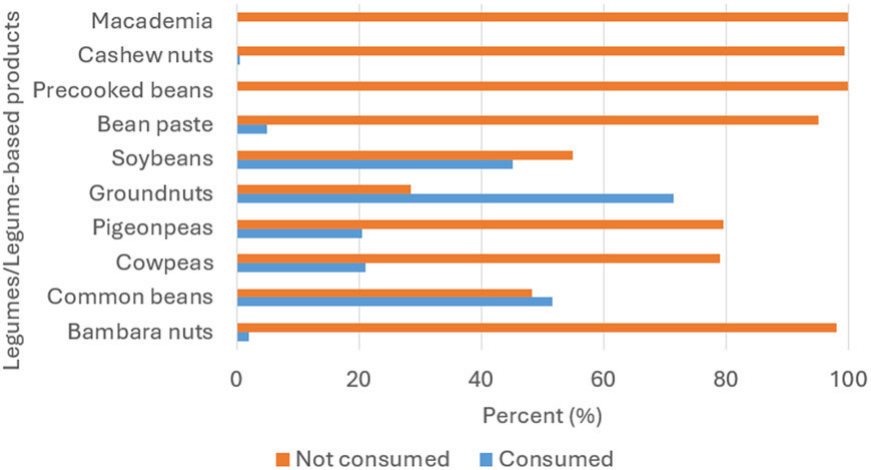
Proportion of children based on their consumption of legumes and legume-based products, showing the dietary contribution of these foods within the study population.

**Figure 4.**
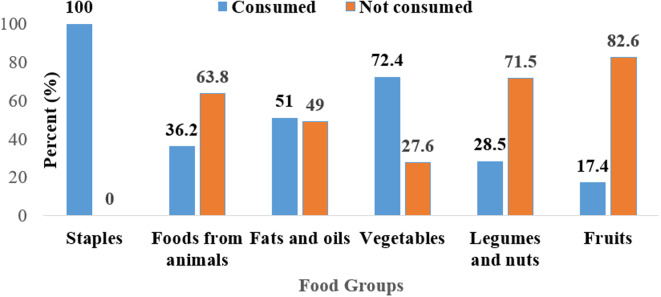
Proportion of children categorised by their consumption of foods from the six Malawian food groups within the 24 hours preceding the survey, illustrating dietary diversity in the study population.

Figure 3 illustrates the percentage of children who consumed legumes and legume-based products in the 24 hours preceding the survey. The study showed revealed that more than 50% of the children had not consumed legumes such as Bambara nuts (98%), cowpeas (79%), pigeon peas (79.5%), soybeans (54.9%), cashew nuts (99.4%) and macadamia (100) the previous day. More than 90% of the children did not consume legume-based products such as precooked beans (95.1%) and bean paste (99.4%). The results show that more than 60% of the children in the study had not consumed foods from legumes and nuts (71.5%), foods from animals (63.8%), and fruits (82.6%). All the children in the study consumed food from staples (100%) while the least (17.4%) consumed fruits (Figure 4).

### Association of food groups and legume consumption with wasting, stunting and underweight

The data in Table 2 shows associations between different food groups and nutritional status indicators (wasting (WHZ), stunting (HAZ), and underweight (WAZ)). Notably, consuming foods from animals was associated with lower odds of underweight (AOR = 0.55, 95% CI: 0.39–0.77, *p* = 0.001) and increased odds wasting (AOR = 3.22, 95% CI: 2.09–4.98, *p* = 0.000). Furthermore, this study showed that consumption of foods from fats and oils was observed to be associated with reduced odds of children from becoming wasting (AOR = 0.45, 95% CI: 0.29–0.70, *p* = 0.000) and stunting (AOR = 0.60, 95% CI: 0.47–0.76), *p* = 0.000). There was no significant association between consumption of vegetables and legumes and nuts with all nutritional status indicators. The results also showed that consumption of fruits by the children was significantly observed to be associated with decreased odds of wasting (AOR = 0.43, 95% CI: 0.22–0.84, *p* = 0.014) and higher odds of underweight (AOR = 2.33, 95% CI: 1.63–3.32, *p* = 0.000).

**Table 2.** Bivariate and multivariate regression analysis of the association between legumes and legume-based foods and wasting, stunting and underweight

Variable	Wasting (WHZ)		Stunting (HAZ)		Underweight (WAZ)	
LEGUMES	AOR (95% CI)	*P*-value	AOR (95% CI)	*P*-value	AOR (95% CI)	*P*-Value
Bambara nuts	2.69(0.87–8.32)	0.086	1.32(0.59–2.96)	0.501	0.57(0.17–1.94)	0.370
Common beans	0.54(0.31–0.95)	0.033*	0.66(0.49–0.88)	0.005*	0.98(0.68–1.41)	0.900
Cowpeas	0.94(0.50–1.77)	0.841	1.55(1.14–2.09)	0.005*	0.99(0.68–1.45)	0.960
Pigeon peas	0.14(0.05–0.41)	0.000*	1.54(1.15–2.07)	0.004*	1.40(0.98–2.00)	0.067
Groundnuts	0.68(0.42–1.12)	0.135	0.88(0.65–1.18)	0.383	1.68(1.11–2.53)	0.013*
Soybeans	2.14(1.27–3.63)	0.005*	1.36(1.03–1.79)	0.031*	1.06(0.75–1.50)	0.754
Bean paste	0.25(0.03–1.90)	0.181	0.70(0.40–1.21)	0.198	0.79(0.39–1.58)	0.497
Precooked beans	1		1		1	
Cashew nuts	1		1		1	
Macademia	1		1		1	

* Statistically significant *P* < 0⋅05.

In contrast, vegetables were observed to be associated with reduced odds of wasting but do not show significant correlations with stunting (AOR = 0.93, 95% CI: 0.72–1.19, *p* = 0.549). The study further revealed that there were no observed significant overall associations between legumes and nuts consumption and nutritional status indicators. However, specific legumes showed different effects: consumption of pigeon peas had protective effect against wasting (AOR = 0.14, 95% CI: 0.05–0.41, *p* = 0.000) but increased the odds of stunting (AOR = 1.54, 95% CI: 1.15–2.07, *p* = 0.004), while consumption of common beans was observed to be associated with decreased odds of both wasting (AOR = 0.54, 95% CI: 0.31–0.95, *p* = 0.033) and stunting (AOR = 0.66, 95% CI: 0.49–0.88, *p* = 0.005). Groundnuts, on the other hand, were observed to be associated with higher odds of underweight (AOR = 1.68, 95% CI: 1.11–2.53, *p* = 0.013).

## Discussion

This study investigated the association between legume consumption and undernutrition among children aged 6–59 months in rural Malawi, with analyses accounting for key feeding status differences captured in the dataset. Children were classified based on whether they were breastfed, non-breastfed, or formula-fed, allowing the model to adjust for variations in energy intake and dietary transitions that influence growth outcomes. After accounting for these feeding patterns, the study revealed high levels of stunting (42.8%), underweight (17.4%), and wasting (8.4%), indicating a persistent challenge of undernutrition in this population. Similar patterns have been reported across sub-Saharan Africa, where stunting remains prevalent due to chronic food insecurity, limited dietary diversity, and poor access to nutrient-rich foods.^(20,21)^

The mean age of children in this study was 28.42 ± 15.10 months, placing many within a critical period of growth where proper nutrition is crucial to prevent irreversible damage to physical and cognitive development.^(22)^ Breastfeeding plays a vital role in shaping a child’s nutrition, especially during the first year of life.^(23)^ In this study, most of the children aged 6–11 months (89.6%) were still being breastfed, while a smaller portion (10.4%) were not. Continuing to breastfeed at this age provides not only essential nutrients but also immune support, helping children grow and develop well, particularly in communities where access to diverse or nutrient-rich complementary foods is limited.^(24,25)^ The high rate of breastfeeding observed in this study reflects both national health recommendations and the strong cultural practices around infant feeding in rural Malawi. On the other hand, children who were not breastfed during this critical period may face higher risks of undernutrition, as formula or early complementary foods might not adequately meet their nutritional needs, especially in low-resource settings.^(26)^ Despite the low incidence of diarrhoea (81.9%) and fever (47.5%) in the two weeks prior to the survey, the high prevalence of stunting suggests that chronic undernutrition may be influenced more by sustained inadequate dietary intake rather than recent illness episodes.^(27)^

Socioeconomic factors such as caregiver education, household income, and occupation emerged as key determinants of child nutrition. Approximately 37.1% of caregivers had attained only junior primary education, which is associated with limited knowledge of proper child feeding practices and the benefits of dietary diversity.^(28)^ Additionally, most households (93.5%) earned less than 50,000 MK ($28.79 USD) per month, reflecting widespread poverty that restricts access to a balanced diet, including protein-rich legumes known to improve nutritional quality.^(29)^ Most caregivers (72.2%) were engaged in farming, which suggests a reliance on subsistence agriculture, leaving these households vulnerable to seasonal fluctuations and climate change impacts that affect crop yields and food availability.^(30)^ Furthermore, the large household sizes, with 59.2% having more than four members, may strain limited resources, reducing the quantity and quality of food available to young children.^(31)^ Addressing these challenges requires targeted interventions, such as nutrition education, support for sustainable agriculture, and programmes to boost household income, to improve child nutritional outcomes and overall resilience.^(32,33)^

The findings of this study highlight a significant gap in the dietary intake of legumes and legume-based products among children aged 6–59 months in rural Malawi. As shown in Figure 3, more than 50% of the children had not consumed legumes such as Bambara nuts (98%), cowpeas (79%), pigeon peas (79.5%), soybeans (54.9%), cashew nuts (99.4%), and macadamia (100%) in the 24 hours preceding the survey. Additionally, more than 90% of the children did not consume legume-based products, such as precooked beans (95.1%) and bean paste (99.4%). This low consumption of legumes is concerning, as legumes are rich sources of protein, iron, and other micronutrients essential for child growth and development.^(34,35)^ Studies have shown that integrating legumes into the diets of young children can significantly improve growth outcomes and reduce the risk of undernutrition.^(34,36)^

Furthermore, the study revealed that over 60% of children did not consume other key food groups such as legumes and nuts (71.5%), foods from animals (63.8%), and fruits (82.6%), as depicted in Figure 4. Limited dietary diversity is a well-documented contributor to micronutrient deficiencies in young children, particularly in rural settings.^(37)^ While all the children consumed food from staples (100%), their heavy reliance on carbohydrate-based foods may contribute to a lack of essential nutrients, thus increasing the risk of malnutrition.^(38,39)^ Additionally, the low fruit consumption (17.4%) suggests a deficiency in vitamin-rich foods, such as vitamin A and vitamin C, which are critical for immune function and growth in children.^(40,41)^ A recent study in sub-Saharan Africa emphasised that diversified diets, including legumes and animal-source foods, are essential to reduce stunting and wasting among young children.^(28)^

These results suggest a need for targeted interventions that promote the consumption of legumes and other protein-rich food groups among children to enhance dietary diversity and nutritional status. Strategies such as community nutrition education programmes and agricultural support aimed at increasing legume production could improve access to these vital food sources.^(42,43)^ Integrating legumes into child feeding programmes and enhancing awareness among caregivers about the nutritional benefits of legumes could play a pivotal role in addressing the dietary gaps identified in this study.^(44,45)^ Such initiatives could significantly improve the nutritional health and developmental outcomes of children in rural Malawi, supporting broader national goals for reducing child malnutrition.^(46)^

The results of this study demonstrate a nuanced and complex relationship between legume consumption and child nutritional outcomes in rural Malawian households, highlighting both positive and potentially adverse effects. Households that consumed common beans and pigeon peas within the 24 hours preceding the survey had lower odds of having wasted children, with odds ratios of 0.54 and 0.14, respectively. This aligns with previous studies showing that legumes, being rich in proteins, dietary fibre, and essential micronutrients like iron and zinc, contribute positively to child growth and development, particularly in preventing wasting, which is often a result of acute malnutrition and insufficient protein intake.^(47,48)^ The high energy density of these legumes also supports children’s caloric needs, which is crucial in settings where malnutrition is prevalent due to limited food availability.^(49)^ Legumes like common beans have been shown to improve nutritional status and prevent wasting due to their high nutrient content and ability to maintain body mass.^(50)^

Interestingly, however, the study revealed that cowpeas and pigeon peas were associated with an increased risk of stunting, with adjusted odds ratios of 1.55 and 1.54, respectively. While legumes are generally regarded as beneficial, their contribution to stunting in this context may be linked to broader dietary factors. If legumes were consumed as staple foods in the absence of complementary nutrient-dense foods, such as animal-source foods, fruits, and vegetables, the overall nutritional intake might have been insufficient to support optimal growth.^(51,52)^ Moreover, antinutritional factors such as phytates in legumes can bind essential minerals like iron and zinc, reducing their bioavailability and potentially contributing to stunting in populations that rely heavily on legumes as their primary protein source.^(53,54)^ Improper preparation and cooking techniques, such as insufficient soaking or fermentation, may also limit nutrient absorption and exacerbate malnutrition outcomes.^(55–58)^

Another notable finding from the study is the increased risk of underweight children in households that consumed groundnuts, with an adjusted odds ratio of 1.68. Groundnuts are high in healthy fats and protein, but in contexts where food diversity is limited, their consumption may not adequately meet the full spectrum of nutritional needs for children.^(44)^ Households that predominantly consume groundnuts may inadvertently reduce their intake of other food groups, leading to imbalances in nutrient intake, particularly if their diets are not diversified with other sources of vitamins and minerals essential for growth.^(59,60)^ Additionally, groundnuts are known to be prone to aflatoxin contamination, which has been linked to stunted growth and underweight children, further complicating their role in child nutrition.^(61,62)^

The study also found that soybean consumption was associated with increased risks of both wasting (AOR = 2.14) and stunting (AOR = 1.36). Soybeans are widely recognised for their high protein content, yet they may not always be consumed in a way that maximises their nutritional benefits.^(63–66)^ In households where soybeans constitute a large part of the diet, the lack of dietary diversity and the reliance on plant-based sources of protein without sufficient complementary foods could exacerbate malnutrition.^(67)^ Additionally, the processing and preparation methods used for soybeans may affect their nutritional value, particularly in reducing antinutritional factors like protease inhibitors and phytates, which impair protein and mineral absorption.^(49,54,68–70)^

### Strengths and limitations

#### Strengths

The study draws on a large, regionally representative sample from three districts in Malawi, improving the reliability and generalisability of the findings. Anthropometric assessments followed standardised WHO procedures, and dietary data were collected using structured 24-hour recall tools to reduce measurement and interviewer errors. Multivariate logistic regression controlled for key demographic and household factors, strengthening confidence in the observed associations.

#### Limitations

The cross-sectional design of the study limits the ability to draw causal conclusions. Dietary intake was captured using a single 24-hour recall without portion-size estimation, which may not reflect usual consumption and is subject to recall and social desirability bias. Interpretation is further constrained by the exclusion of incomplete cases and the absence of biochemical measures such as micronutrient markers or aflatoxin exposure. Additional unmeasured factors including seasonal food availability, illness, and variations in legume preparation may also have influenced the findings.

## Conclusion

This study highlights the important link between legume consumption and undernutrition in children aged 6–59 months in rural Malawi. The results show significant gaps in the intake of legumes, with more than 50% of children not eating key legumes such as Bambara nuts, cowpeas, pigeon peas, and soybeans in the past 24 hours. This low consumption is troubling, as legumes are essential sources of protein and micronutrients crucial for growth and development. Additionally, over 60% of children did not consume other vital food groups, such as animal-source foods and fruits, which worsens dietary diversity and can increase the risk of malnutrition.

The study also reveals a complex relationship between legume consumption and child nutritional outcomes. While eating common beans and pigeon peas is linked to a lower risk of wasting, cowpeas, pigeon peas, and soybeans are associated with higher risks of stunting and wasting. These findings indicate that the benefits of legumes can vary depending on the overall diet, especially in households with limited access to a variety of nutrient-rich foods.

## Data Availability

Data from this study will be made available upon request from the corresponding author.
